# Surgical Resection of Primary Tumors Provides Survival Benefits for Lung Cancer Patients With Unexpected Pleural Dissemination

**DOI:** 10.3389/fsurg.2021.679565

**Published:** 2021-06-23

**Authors:** Liwen Fan, Haitang Yang, Ke Han, Yang Zhao, Wen Gao, Ralph A. Schmid, Feng Yao, Heng Zhao

**Affiliations:** ^1^Department of Thoracic Surgery, Huadong Hospital Affiliated to FuDan University, Shanghai, China; ^2^Department of Thoracic Surgery, Shanghai Chest Hospital, Shanghai Jiao Tong University, Shanghai, China; ^3^Department of General Thoracic Surgery, Department of BioMedical Research, Inselspital, Bern University Hospital, University of Bern, Bern, Switzerland

**Keywords:** non-small cell lung cancer, malignant pleural nodule, surgery, epidermal growth factor receptor, survival

## Abstract

**Background:** Surgery is not generally recommended for non-small cell lung cancer (NSCLC) patients with malignant pleural dissemination (PD). However, in some cases, PD is found unexpectedly during surgery. There is no consensus on whether surgical intervention can provide survival benefit for them. We investigated the role of surgery in NSCLC patients with unexpected PD by a cohort study.

**Methods:** Clinical data of consecutive patients who intended to undergo radical surgery for NSCLC between January 2010 and December 2015 at Shanghai Chest Hospital and Huadong Hospital were collected from a lung cancer database. Patients diagnosed with unexpected malignant pleural nodules intraoperatively were enrolled in this retrospective study.

**Results:** A total of 181 NSCLC patients were diagnosed with unexpected malignant PD intraoperatively and confirmed with postoperatively histological examinations. Out of these, 80 (44.2%) patients received pleural nodule biopsies alone, and 101 (55.8%) received primary tumor resection (47 with sublobar resection and 54 with lobectomy). The median progression-free survival and overall survival for all patients were 13 and 41 months respectively. Patients in the resection group had significantly better progression-free survival (19.0 vs. 10.0 months, *P* < 0.0001) and overall survival (48.0 vs. 33.0 months, *P* < 0.0001) than patients in the biopsy group. In the resection group, there was no statistical difference between patients with sublobar resection and lobectomy (*P* = 0.34). Univariate and multivariate analyses identified primary tumor resection, targeted adjuvant therapy, and tumor size (≤ 3 cm) as independent prognostic factors.

**Conclusions:** NSCLC patients with unexpected intraoperative PD potentially benefited from surgical resection of the primary tumor and multidisciplinary targeted therapy, particularly when tumor size did not exceed 3 cm. Our data demonstrated that the resection type was not associated with survival differences, which remains to be defined with a larger sample size.

## Introduction

About 4.5–7.5% of patients with non-small cell lung cancer (NSCLC) are confirmed with pleural dissemination (PD) at diagnosis (1, 2). NSCLC with PD is typically staged as M1a in the 7th and 8th tumor, node, and metastasis (TNM) classification because NSCLC patients with PD had a generally poor prognosis (3–5). The median overall survival (OS) and 5-year survival rate were 8 months and <2%, respectively (2).

Because NSCLC with PD are classified as M1a stage, thus, systemic chemotherapy or targeted therapy, rather than surgical resection, is recommended as standard care for patients at initial diagnosis (6). However, sometimes PD is found unexpectedly during operation. In this case, it is difficult for surgeons to determine whether to proceed the resection of the primary tumors or not, given that, on one hand, there is a lack of evidence of surgical role in unexpected PD cases due to the low incidence of unexpected PD cases, and, on the other hand, there is no technical difficulty with surgical excision of primary and metastatic pleural lesions. Furthermore, with the rapid development of targeted drugs, multidisciplinary treatment including surgery may improve the survival of PD patients bearing a sensitive mutation. But relevant studies focusing on targeted therapy are limited.

In recent years, it was reported that surgical resection showed prognosis benefits for NSCLC with malignant PD (1, 7–13). Several studies focused on patients with PD showed good survival after tumor resection, but without statistical difference or control group (1, 14–17). Besides, some studies included patients with pleural effusion >100 ml in the cohort, which could be found preoperatively and was a sign for metastasis, leading to a potential bias for survival analysis. (7, 16, 18, 19). Li et al. (12) and Ren et al. (7) reported that surgical resection was the significant prognostic factor of patients with unexpected PD, but the sample size was small (43–83 cases), which reduced the level of evidence. On the contrary, a study by Sawabata indicated that tumor resection was not beneficial for the survival (19). Still, the role of surgery in NSCLC with PD remains controversial.

Given the limited evidence concerning the role of surgical resection in NSCLC patients with unexpected PD detected during surgery, we conducted a retrospective study with larger sample size to analyze the clinical characteristics, pathological features, positive mutations and prognosis of patients who intended to undergo surgery and were unexpectedly found to have intraoperative malignant pleural nodules (MPN).

## Materials and Methods

### Patients Demographics

Clinical data of 21,591 consecutive patients who intended to undergo radical surgery for NSCLC between January 2010 and December 2015 at Shanghai Chest Hospital and Huadong Hospital were collected. The patients with unexpected PD, Eastern Cooperative Oncology Group performance status (PS) of 0 to 1 were enrolled in this study. Unexpected PD is defined as (1) preoperative assessments did not detect PD or distant metastasis; (2) no malignant pleural effusion was found preoperatively; (3) PD was only accidentally identified during operations; (4) postoperative pathology confirmed the tumor dissemination to pleural. PD could be separated into localized MPN (several local nodules which could be resected by limited resection) or diffused MPN (uncountable nodules distributed over the parietal pleura).

Clinical characteristics of the patients and respective tumors were abstracted from the electronic medical records by professional staff. NSCLC staging was performed according to the 8th TNM classification (5). Given that the pathological information is incomplete in a considerable number of patients, for example, patients receiving biopsy only or undergoing resection but without systemic lymph node dissection, thus the concept of the best stage instead of the pathological stage was used, which was based on the pathological stage if available, otherwise, the clinical stage would be used instead. Meanwhile, considering the inaccuracy of the stage information, it was excluded in the analysis of prognostic factors. This study was approved by the committees for ethical review of research at Shanghai Chest Hospital and Huadong Hospital, and informed consent was not required because of the retrospective nature of it.

### Clinical Assessments

All patients underwent thorough preoperative evaluations preoperatively, including physical examination, routine laboratory tests, serum tumor markers (carcinoembryonic antigen, cancer antigen 125, neuron-specific enolase, cyfra21-1, squamous cell carcinoma antigen), chest computed tomography (CT), respiratory function test, echocardiography, and electrocardiogram. Distant or extrathoracic metastasis was excluded by brain magnetic resonance imaging (MRI), abdominal CT or sonography, and bone scanning. Positron emission tomography (PET) was applied if applicable.

### Operations

Posterolateral thoracotomy or video-assisted thoracoscopic surgery (VATS) was performed by surgeons according to the patient's conditions. When pleural nodule was found during the initial exploration, a frozen section biopsy of the pleural was taken to confirm the pleural metastasis. Then different types of surgical resections were chosen by surgeons based on their experience, beliefs, and conditions of patients. These included pleural nodules biopsy, primary tumor resection (wedge resection, segmentectomy, or lobectomy) with or without systemic lymphadenectomy, lymph node sampling, pleurectomy, or pleural nodule resection or electrocautery.

### Follow-Up

All patients were instructed to receive 4–6 cycles of adjuvant platinum-based chemotherapies or first-line targeted therapies if they harbored sensitive mutations for medications. Radiotherapies were performed for local progression or distant metastasis, according to the radiation oncologists. Adjuvant therapies were prescribed within 1 month postoperatively.

The follow-up visit was scheduled as the National Comprehensive Cancer Network (NCCN) guidelines (6). All patients were evaluated by a chest CT scan and abdominal sonography. Additionally, brain MRI and bone scintigraphy were regularly performed according to the physicians when necessary. When patients suffered disease progression, subsequent chemotherapy, or targeted therapy was recommended based on the suggestions by oncologists.

OS and progression-free survival (PFS) were regarded as the primary endpoints of the study. OS was recorded from the date of surgery to the date of death or the last follow-up visit. PFS was measured from the date of surgery until the date of the first documented progression or the last follow-up. The closing date of the follow-up for this study was January 31, 2018. Information was obtained from patients through phone calls and outpatient re-visit records.

### Statistical Analysis

Measurement data were assessed to compare different patient groups by the chi-square test and Fisher exact probability test for categorical variables and two-tailed Student's *t*-test for continuous variables. And continuous variables were summarized as median and range. Categorical variables were expressed by the median and percentage. Survival curves were obtained using Kaplan-Meier method and compared using the log-rank test. Univariate and multivariate analysis use the Cox proportional hazards regression with the test level α = 0.05. The proportional hazard assumption was examined and met by plotting the survival curve with Kaplan-Meier method. Significant variables in univariate analysis (defined as *P* < 0.15) would be included in multivariate analysis. Other clinically relevant factors like sex, age, and smoking history were also included in the Cox proportional-hazards model (20). A *P*-value of < 0.05 was considered statistically significant. All analyses were conducted using SPSS 22.0 software (IBM Corporation, Chicago, Illinois, USA) and R software (version 3.6.3).

## Results

### Patients Clinicopathological Characteristics

Two hundred seventeen (1.0%) of 21,591 cases were found to have a PD. After the exclusion of 36 patients not meeting the inclusion criteria, a total of 181 patients (98/54.1% men, 83/45.9% women) diagnosed with unexpected malignant PD through intraoperatively or postoperatively histological examinations were enrolled in the present study. The median age of them was 59 years ranging 30–75 years. The characteristics of patients and tumors were summarized in Table 1. The median follow-up duration was 36 months (range, 4–90 months). Thirteen (7.2%) patients were lost to follow-up. Therefore, 168 patients were included in the survival analysis. Thirty-nine of 181 (21.5%) patients received PET, and no evidence for PD was found preoperatively.

**Table 1:** Baseline clinical features of pleural biopsy group and primary tumor resection group.

**Variables**	**Total (*n* = 181)**	**Biopsy group (*n* = 80)**	**Resection group (*n* = 101)**	***P-*value**
	**(count, %)**	**(count, %)**	**(count, %)**	
Age (years, median, range)	59, 30–75	60, 35–75	57.5, 30–75	0.39
Gender				0.34
Male	98 (54.1)	40 (50)	58 (57.4)	
Female	83 (45.9)	40 (50)	43 (42.6)	
Location				0.77
Right	110 (60.8)	48 (60.0)	62 (61.4)	
Left	71 (39.2)	32 (40.0)	39 (38.6)	
Smoking history				0.89
Yes	62 (34.3)	27 (33.8)	35 (34.7)	
No	119 (65.7)	53 (66.2)	66 (65.3)	
Performance status				0.31
0	141 (77.9)	59 (73.8)	82 (81.2)	
1	40 (22.1)	21 (26.2)	19 (18.8)	
Tumor location types				0.189
Central	29 (16.0)	16 (20.0)	13 (12.9)	
Peripheral	152 (84.0)	64 (80.0)	88 (87.1)	
Chemotherapy				0.58
Yes	163 (90.1)	73 (91.2)	90 (89.1)	
No	18 (9.9)	7 (8.8)	11 (10.9)	
Targeted therapy				0.11
Yes	99 (54.7)	38 (47.5)	61 (60.4)	
No	61 (33.7)	29 (36.3)	32 (31.7)	
Unknown	21 (11.6)	13 (16.2)	8 (7.9)	
EGFR mutation				0.35
Yes	65 (35.9)	21 (26.2)	44 (43.6)	
No	50 (27.6)	20 (25.0)	30 (29.7)	
Unknown	66 (36.5)	39 (48.8)	27 (26.7)	
Radiotherapy				0.15
Yes	56 (30.9)	22 (27.5)	34 (33.7)	
No	88 (48.6)	36 (45.0)	52 (51.5)	
Unknown	37 (20.4)	22 (27.5)	15 (14.8)	

*EGFR, epidermal growth factor receptor*.

### Surgery

Of the 181 patients, 100 (55.2%) patients underwent VATS procedure, and 81 (44.8%) had thoracotomy. Eighty (44.2%) received pleural nodule biopsy alone (biopsy group), and 101 (55.8%) underwent additional primary tumor resection (resection group). In the resection group, 47 (46.5%) cases received sublobar resection (segmentectomy in 7 cases and wedge resection in 40 cases), and 54 (53.5%) cases had lobectomy. Systemic lymphadenectomy and lymph node sampling were performed in 45 and 26 cases, respectively. Additionally, 43 (23.8%) patients were detected with localized MPN intraoperatively, while 138 (76.2%) had diffused MPN (Table 2). No severe intraoperative and postoperative complications occurred. There was no postoperative mortality in neither group.

**Table 2:** Operative and pathological findings of pleural biopsy group and primary tumor resection group.

**Variables**	**Total (*n* = 181)**	**Biopsy group (*n* = 80)**	**Resection group (*n* = 101)**	***P-*value**
	**(count, %)**	**(count, %)**	**(count, %)**	
Surgery method				<0.001^*^
Biopsy alone		80 (100.0)	0 (0)	
Sublobar resection		0 (0)	47 (46.5)	
Wedge resection		0 (0)	40	
Segmentectomy		0 (0)	7	
Lobectomy		0 (0)	54 (53.5)	
Surgical approach				0.06
VATS	100 (55.2)	51 (63.8)	49 (48.5)	
Thoracotomy	81 (44.8)	29 (36.2)	52 (51.5)	
Lymph node resection				<0.001^*^
No	110 (60.8)	69 (86.3)	41 (40.6)	
Lymph node sampling	26 (14.4)	11 (13.7)	15 (14.8)	
Lymphadenectomy	45 (24.8)	0 (0)	45 (44.6)	
Operation duration (minutes, median, range)	75, 15–230	61, 25–149	91.5, 15–230	<0.001^*^
Operative bleeding (ml)				0.002^*^
≤ 100	132 (72.9)	68 (85.0)	64 (63.4)	
>100	49 (27.1)	12 (15.0)	37 (36.6)	
Postoperative hospitalization (days, median, range)	5, 1–22	4.5, 1–22	6, 1–15	0.008^*^
Pathological type				0.13
Adenocarcinoma	155 (85.6)	65 (81.3)	90 (89.1)	
Others	26 (14.4)	15 (18.7)	11 (10.9)	
Tumor size (cm, median, range)	3.2, 0.7–9.0	3.75, 1.2–8.0	3.0, 0.7–9.0	0.004^*^
Malignant pleural nodule				<0.001^*^
Localized	43 (23.8)	9 (11.2)	34 (33.7)	
Diffused	138 (76.2)	71 (88.8)	67 (66.3)	
Best T stage				0.112
T1	13 (7.2)	3 (3.7)	10 (9.9)	
T2	80 (44.2)	27 (33.7)	53 (52.5)	
T3	33 (18.2)	17 (21.3)	16 (15.8)	
T4	39 (21.6)	18 (22.5)	21 (20.8)	
Tx	16 (8.8)	15 (18.8)	1 (1.0)	
Best N stage				<0.001^*^
N0	50 (27.6)	8 (10.0)	42 (41.6)	
N1	26 (14.4)	13 (16.3)	13 (12.9)	
N2	66 (36.5)	29 (36.3)	37 (36.6)	
N3	8 (4.4)	7 (8.7)	1 (1.0)	
Nx	31 (17.1)	23 (28.7)	8 (7.9)	

*VATS, video-assisted thoracoscopic surgery*.

* *The bold values represented the statistically significant values*.

### Pathology

Pathologic types of these patients included adenocarcinoma (155; 85.6%), squamous cell carcinoma (13; 7.2%), adenosquamous cell carcinoma (5; 2.8%), large cell carcinoma (3; 1.6%), and others (5; 2.8%). Adenocarcinoma was the predominant pathological type in both the biopsy and resection groups. The patients with the T2 or N2 stage were in the majority, accounting for 44.2 and 36.5%, respectively (Table 2). Additionally, sensitive mutations were examined for targeted therapies after surgery, and positive results were found which included epidermal growth factor receptor (EGFR) mutations in 65 of 115 (56.5%) cases (38 with a deletion in exon 19, 27 with a point mutation at codon 858 in exon 21, and 1 with an insertion mutation in exon 20), and anaplastic lymphoma kinase (ALK) rearrangement in 12 of 63 (19.0%) cases.

### Adjuvant Therapies

One hundred and thirty nine (82.7%) patients undertook platinum-based chemotherapies as first-line treatment, of which three patients received sequential EGFR-TKIs. Twenty-seven patients undertook targeted therapy as first-line treatment with EGFR-TKIs, including gefitinib (Iressa), erlotinib (Tarceva), and icotinib (Conmana) and anaplastic lymphoma kinase tyrosine kinase inhibitor (ALK-TKI) (crizotinib, Xalkori). After disease recurrence was detected, second-line chemotherapies or targeted therapies were prescribed. In total, 99 of 160 (61.9%) patients received TKIs postoperatively (Table 1). Particularly, those EGFR-mutant patients harboring EGFR substitution of threonine 790 with methionine (T790M) received osimertinib (AZD9291) after drug resistance of former EGFR-TKIs in 15 cases. Radiotherapy was administered in 56 patients for local control or metastasis.

### Survival

The median PFS and median OS were 13 months and 41 months, respectively. The 3- and 5-year PFS and survival rate for all patients were 13.1%, 5.7%, and 56.0%, 28.7%, respectively.

#### Clinicopathological Characteristics and Survival Comparison of Patients in the Biopsy and Resection Group

There is no significant difference concerning the baseline characteristics of patients (Table 1). However, the resection group has significantly smaller tumor size (median 3.0 vs. 3.75 cm; *P* = 0.004) and less cases with diffused MPN (67/66.3% vs. 71/88.8%; *P* < 0.001) than those in the biopsy group. Additionally, in the resection group, the operation duration (median 91.5 vs. 61 min; *P* < 0.001) and postoperative hospital stay (median 6 vs. 4.5 days; *P* = 0.008) were significantly longer, and there were more cases of bleeding during the operation (37/36.6% vs. 12/15.0%; *P* = 0.002) (Tables 1, 2).

**Figure 1:**
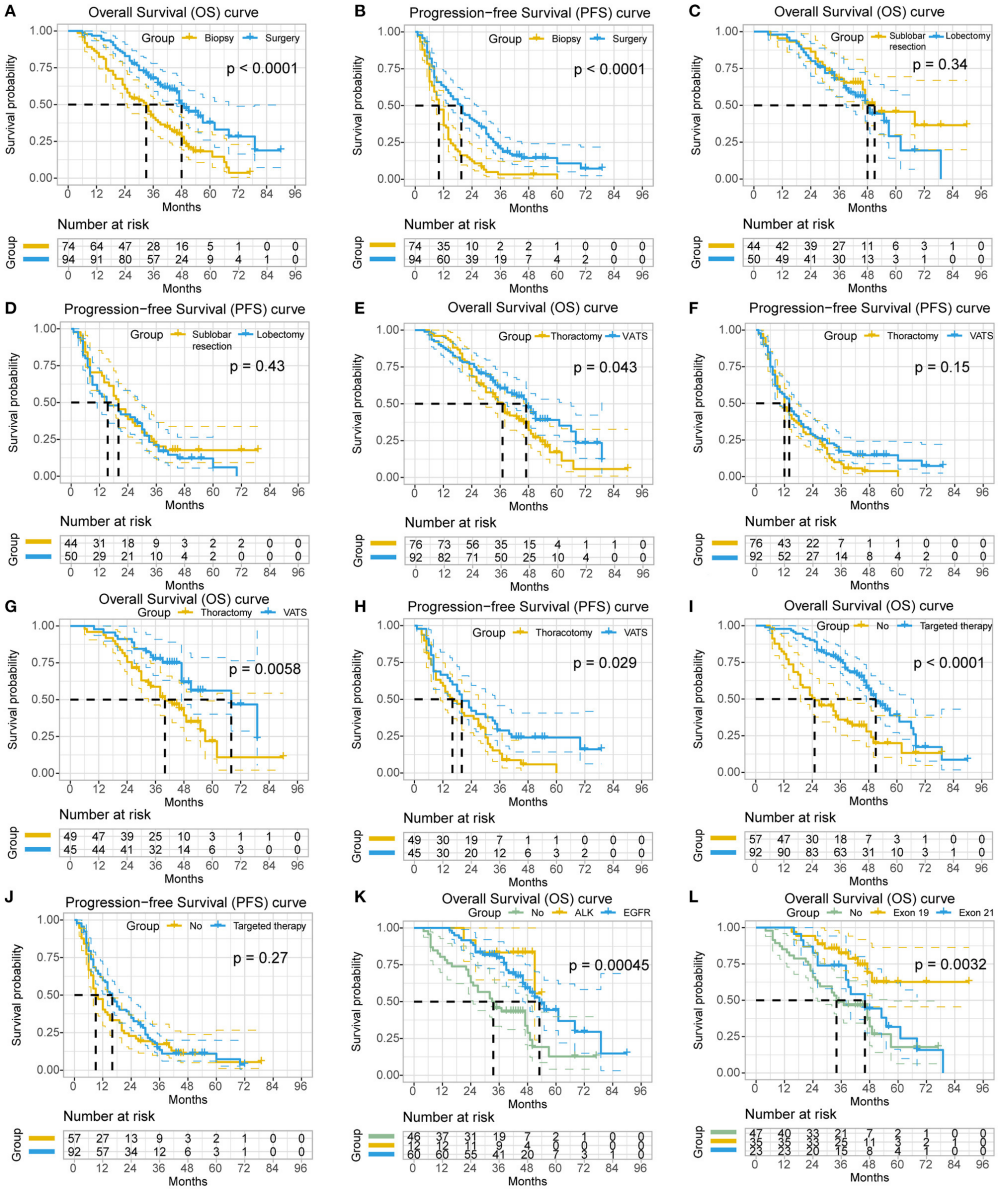
The survival analyses of the clinicopathological characteristics in non-small cell lung cancer (NSCLC) patients with unexpected pleural dissemination (PD). **(A,B)** Comparison of postoperative overall survival (OS) and progression-free survival (PFS) of NSCLC patients with unexpected PD in primary tumor resection group and biopsy alone group. **(C,D)** Comparison of OS and PFS of NSCLC patients with unexpected PD in sublobar resection group and lobectomy group. **(E,F)** Comparison of OS and PFS of NSCLC patients with unexpected PD in video-assisted thoracic surgery (VATS) group and thoracotomy group. **(G,H)** Subgroup analysis of OS and PFS of NSCLC patients with unexpected PD in resection group with or without VATS. **(I,J)** Comparison of OS and PFS of NSCLC patients with unexpected PD receiving targeted therapy or not. **(K)** Comparison of OS of NSCLC patients with unexpected PD having anaplastic lymphoma kinase (ALK) rearrangement, epidermal growth factor receptor (EGFR) mutation, or not. **(L)** Comparison of OS of NSCLC patients with unexpected PD having different subtypes of EGFR mutation.

In comparison, patients in the resection group had significant better PFS [19.0 (95% CI: 14.7–23.3) vs. 10.0 (95% CI: 8.0–12.0) months; *P* < 0.0001] and OS [48.0 (95% CI: 41.5–54.5) vs. 33.0 (95% CI: 25.0–41.0) months; *P* < 0.0001] than those in the biopsy group (Figures 1A,B). The 3- and 5-year PFS rate of the resection group were higher than the biopsy group (20.8% and 10.8% vs. 3.2% and 0%, respectively). Similar results were seen for OS (67.8% and 37.7% vs. 41.0% and 18.2%, respectively). Additionally, subgroup analysis showed that surgical resection still benefited survival significantly (47.0 vs. 19.0 months, *P* < 0.0001) in patients who did not receive targeted therapies.

#### The Role of Surgical Resection, Status of MPN, and Adjuvant Therapies

In the resection group, the 5-year survival rate and OS of patients who underwent sublobar resection were 45.6% and 51.0 months (95% CI 33.6–68.4), respectively, while those of patients underwent lobectomy were 29.1% and 48.0 months (95% CI 39.9–56.1). There was no statistical difference in these different types of surgical resection (*P* = 0.34), although the survival of patients with sublobar resection tended to be better. A similar result was observed for PFS (sublobar resection vs. lobectomy, 20.0 vs. 15.0 months; *P* = 0.425) (Figures 1C,D). Notably, Patients underwent lobectomy had a significantly larger tumor size (*P* < 0.001) and less diffused MPN (48% vs. 84.1%, *P* < 0.001), and more patients with lobectomy underwent thoracotomy (76% vs. 25%, *P* < 0.001) and lymph node resection (96% vs. 18.2%, *P* < 0.001) than patients with sublobar resection. Patients with resection of the primary tumor, either sublobar resection or lobectomy, had better survival than patients undergoing biopsy alone (*P* < 0.001 and *P* = 0.003, respectively). Additionally, in the resection group, no statistical difference was observed regarding the OS of patients with systemic lymphadenectomy, lymph node sampling and no lymph node resection (*P* = 0.380).

With regard to the surgical approaches, patients underwent VATS showed significantly better prognosis than patients underwent thoracotomy (median OS, 47.0 vs. 37.0 months; *P* = 0.043). Subgroup analysis demonstrated a similar result in the resection group (median OS, 68.0 vs. 40.0 months; *P* = 0.006; median PFS, 20.0 vs. 16.0 months; *P* = 0.029) (Figures 1E–H), while no survival difference was observed in the biopsy group (median OS, 33.0 vs. 27.0 months; *P* = 0.484).

No significant survival benefit was observed concerning the amount of MPN in patients within groups (localized vs. diffused, median OS, 46.0 vs. 39.0 months; *P* = 1.0) or subgroups (biopsy group, *P* = 0.667; resection group, *P* = 0.082).

Patients who received targeted therapies had significantly better survival than those not (median OS, 51.0 vs. 25.0 months; *P* < 0.0001) (Figures 1I,J), as well as patients with positive EGFR mutations (median OS, 53.0 vs. 34.0 months; *P* = 0.005). Subgroup analysis also showed a better survival for patients with targeted therapies (median OS, biopsy group, 49.0 vs. 19.0 months, *P* < 0.001; resection group, 55.0 vs. 47.0 months, *P* = 0.034). Additionally, the patients with ALK rearrangement had no survival difference with patients with EGFR mutation [mean OS, 48.1 (95% CI: 41.7–54.5) vs. 55.7 (95% CI: 48.1–63.2) months; *P* = 0.367], but they both had significantly longer survival than patients without mutation [mean OS, 36.2 (95% CI: 29.2–43.3) months; *P* = 0.011 or *P* = 0.001, respectively] (Figure 1K). As for the subtypes of EGFR mutation, patients with a deletion in exon 19 had significantly better survival than patients with a point mutation in exon 21 [mean OS, 69.7 (95% CI: 59.2–80.1) vs. 47.4 (95% CI: 38.5–56.3) months; *P* = 0.024] (Figure 1L).

#### Risk Factors for Prognosis

**Figure 2:**
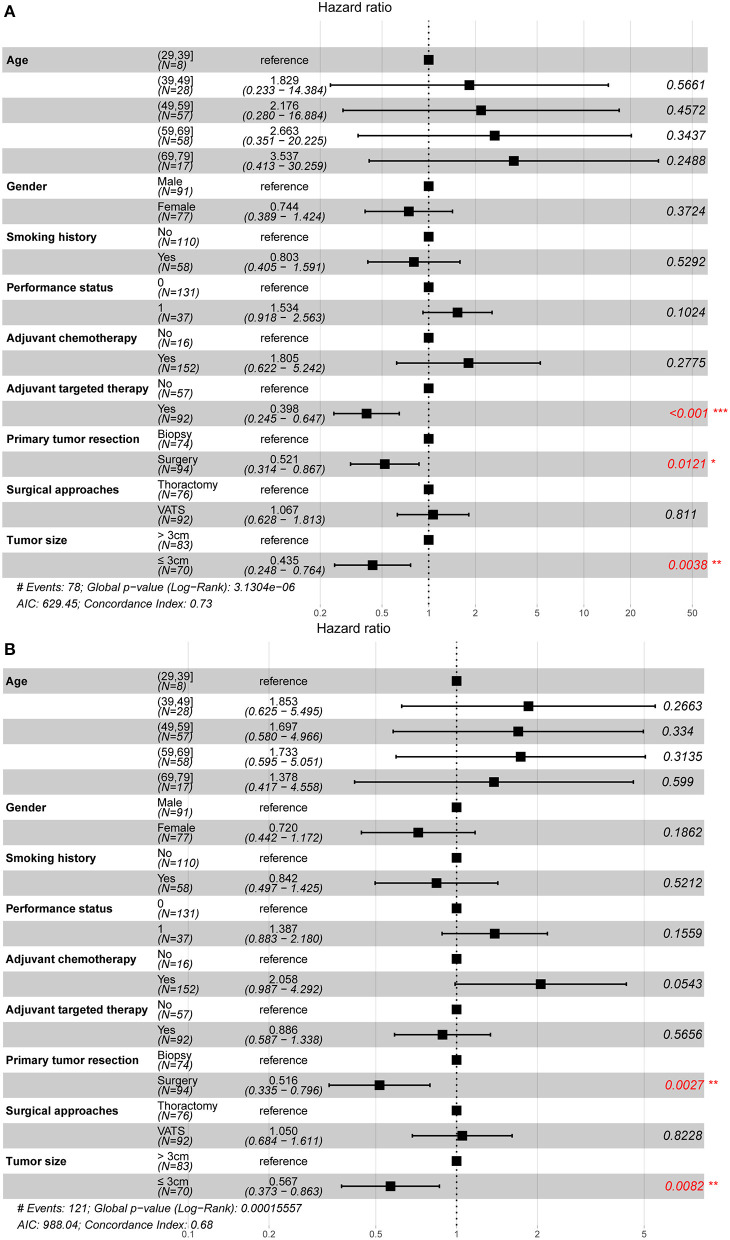
Prognostic factors of non-small cell lung cancer patients with unexpected pleural dissemination after surgery. **(A)** The forest plot of multivariate Cox regression analysis for overall survival. **(B)** The forest plot of multivariate Cox regression analysis for progression-free survival. VATS, video-assisted thoracic surgery. *statistically significant.

Univariate (Table 3) and multivariate (Figure 2) analyses indicated that primary tumor resection [hazard ratio (HR) 0.52, 95% CI 0.31–0.87, *P* = 0.012], adjuvant targeted therapy (HR 0.40, 95% CI 0.25–0.65, *P* < 0.001), and tumor size ≤ 3 cm (HR 0.44, 95% CI 0.25–0.76, *P* = 0.004) were associated with increased OS, while primary tumor resection (HR 0.52, 95% CI 0.34–0.80, *P* = 0.003) and tumor size ≤ 3 cm (HR 0.57, 95% CI 0.37–0.86, *P* = 0.008) were associated with increased PFS among patients with unexpected PD. Additionally, subgroup analysis in resection group demonstrated that resection type was not associated with better survival by Cox regression model (HR 1.323, 95% CI 0.743–2.357, *P* = 0.342).

**Table 3:** Univariate analysis of prognostic factors.

**Variables**	**Progression-free survival**		**Overall survival**	
	**Hazard ratio (95%CI)**	***P-*value**	**Hazard ratio (95%CI)**	***P* value**
Age				
>59 y vs. ≤ 59 y	1.099 (0.799–1.513)	0.561	1.599 (1.091–2.344)	0.015^*^
Gender				
Female vs. Male	0.894 (0.650–1.230)	0.493	0.873 (0.596–1.277)	0.482
Smoking history				
Yes vs. No	1.086 (0.779–1.513)	0.626	1.079 (0.731–1.592)	0.702
Performance status				
1 vs. 0	1.730 (1.190–2.513)	0.004^*^	2.028 (1.316–3.115)	0.001^*^
Adjuvant chemotherapy				
Yes vs. No	2.242 (1.212–4.149)	0.008^*^	2.028 (0.889–4.630)	0.086
Adjuvant targeted therapy				
Yes vs. No	0.827 (0.583–1.174)	0.287	0.416 (0.274–0.632)	<0.001^*^
Primary tumor resection				
Surgery vs. Biopsy	0.478 (0.343–0.665)	<0.001^*^	0.458 (0.312–0.672)	<0.001^*^
Surgical approaches				
VATS vs. Thoracotomy	0.794 (0.576–1.094)	0.159	0.678 (0.463–0.993)	0.046^*^
Tumor size				
≤ 3 cm vs. >3 cm	0.536 (0.380–0.758)	<0.001^*^	0.462 (0.301–0.709)	<0.001^*^
Malignant pleural nodule				
Localized vs. diffused	0.824 (0.554–1.227)	0.340	0.886 (0.621–1.510)	0.968

*CI, confidential interval; VATS, video-assisted thoracoscopic surgery*.

* *The bold values represented the statistically significant values*.

## Discussion

According to the criteria of 7th and 8th lung cancer TNM staging (4, 5), NSCLC with PD is classified as stage IV (M1a), due to which patients with PD are not recommended for the surgical invention. However, in the clinic, surgeons are sometimes faced with the unexpected PD detected during surgery, which is unidentifiable in preoperative examinations, or suspected pleural nodules, which could not be verified due to the lack of histological evidence. Under these circumstances, surgeons have to decide to stop surgery or go on. Recently, emerging evidence have shown that primary tumor resection could provide survival benefits for NSCLC with malignant PD (1, 7–12, 21, 22), which promoted the re-evaluation of the surgical roles in this type of disease, particularly with the rapid development of VATS with less postoperative pain, shorter hospital stays, and fewer complications (23). However, some of the studies have the limitation of small sample size, patient selection bias (containing patients with pleural effusion, contralateral nodules, or no pathological confirmation of PD), or lack of control group (1, 7, 12, 14–19). Taken together, more studies are needed to investigate the value of surgical treatment for patients with unexpected intraoperative PD (13).

According to the previous studies, the OS, 3- and 5-year survival rate of NSCLC patients with PD were 15–52 months, 25.2–69.2% and 16.0–42.7%, respectively (1, 7–9, 11, 17, 21, 22, 24). Furthermore, the OS, 3- and 5-year survival rate were 20–64 months, 45.8–82.9%, and 31.4–42.7% for patients with primary tumor resection, while 7–35 months, 11.8–41.7%, and 0–19.5% for patients with biopsy alone. Our results were similar to these favorable clinical outcomes. Even the outcomes of the biopsy group in this study were better than the previous clinical data (5-year survival rate, 2%; median OS, 9.5–11.5 months), which supports the opinion that unexpected intraoperative PD may belong to a relatively earlier stage than clinical diagnosed PD (5, 6, 10, 25).

PD represents a wide range of disease states from a single metastasis nodule to diffused pleural nodules involving in pericardium and diaphragm with a large amount of pleural effusion. The tumor burden of these states is different. On the one hand, with the application of high-resolution CT and PET, most PD cases could be diagnosed before surgery. So those unexpected intraoperative PD cases were in the relatively early stage of M1a. Ren et al. pointed out the same thesis as well (7). On the other hand, according to the NCCN guidelines for oligometastatic NSCLC (M1b), surgical resection of primary lesion and metastasis can be beneficial for these patients (6). Theoretically, the tumor burden of PD (M1a) is less than M1b, hence the surgery may also be of advantage to the unexpected PD patient's survival. In this study, the results did show a survival benefit for unexpected PD patients from surgery with a median OS of 41 months. Consistent with our outcomes, a Japanese study of 313 NSCLC patients with PD demonstrated that patients underwent macroscopic complete resection had better survival than patients with exploratory thoracotomy (11). Also, Shen et al. (10) reported a retrospective study of patients with stage M1a NSCLC, in which the patients who underwent primary tumor resection had a significantly better OS than patients accepted no surgery or only metastatic tumor resection (*P* < 0.001).

Another possible reason for the favorable results is related to adjuvant therapies, especially targeted therapy. In our study, the majority of patients (166 of 168, 98.8%) underwent postoperative adjuvant therapies. The significantly better survival was observed in patients who received postoperative targeted therapy than those who did not, which suggests that NSCLC patients with unexpected PD can benefit from mutation tests and targeted therapies (11). In the context of the rapid development of anti-tumor drugs, it may be possible to be more active in surgical treatment when encountering unexpected PD.

As for the types of surgical resections, no statistical difference in survival between sublobar resection and lobectomy was observed, similar results were found in some previous studies (7, 8, 10, 12, 17, 24). Notably, the heterogeneity between subgroups of lobectomy and sublobar resection may influence the conclusion, making it difficult to determine which was the best type of resection. Okamoto and Ohta both found that patients received pneumonectomy had a significantly worse survival than patients with limited resection (1, 15). However, a study conducted by Iida et al. showed an opposite view that the survival for patients with macroscopic complete resection was statistically better than patients with macroscopic incomplete resection (*P* = 0.009) and exploratory thoracotomy (*P* < 0.001) (11). Moreover, no statistical difference in survival was observed between patients who underwent systemic lymphadenectomy and not (*P* = 0.29) in our study. The study by Ren et al. also indicated that neither systemic lymphadenectomy nor pleurectomy made difference in survival significantly (7). In summary, more evidences are required to clarify the optimum resection type in these patients, although surgical intervention seems more like a cytoreductive surgery in the PD cases. Sublobar resection by video-assisted thoracoscopic surgery may be a proper choice for the less invasiveness.

Our data demonstrated that patients underwent VATS had a significant better survival, compared with those underwent thoracotomy. However, the multivariate analysis did not support this result. Therefore, the survival difference may be caused by potential bias. Although with the advances of minimally invasive surgery, including robotic-assisted thoracoscopic surgery, better surgical outcomes may improve the patients' quality of life. Additionally, the extent of pleural diffusion is a complex variable, which is difficult to analyze due to the ambiguous definition and limited sample size, and may have a potential influence on the prognosis. Li et al. defined diffused pleural nodules as more than three pleural nodules, and their results showed diffused MPN had no survival difference with localized MPN (11, 12). In our study, there was no significant survival difference between localized MPN and diffused MPN among all patients or in the resection group, although the biopsy group had more patients with diffused MPN. This indicated that patients with less MPN tended to be selected for resection. It needs more research to clarify the prognosis influence of the MPN.

In this study, univariate and multivariate analyses demonstrated that primary tumor resection, targeted adjuvant therapy, and tumor size ≤ 3 cm were independent predictors of survival in NSCLC patients with unexpected PD. These may suggest that surgeons select unexpected PD patients with small tumor size for resection and perform sensitive mutation detection for targeted therapy, routinely. The previous studies have reported similar prognosis factors to ours (7, 12, 21, 26). However, the time span of this study was relatively short and recent (6 years from 2010) compared with other studies (1, 16, 17, 19, 24), and no patient was lack of pathological confirmation for malignant PD, unlike other studies (16, 19). Besides, the sample size of this study was relatively larger than previous studies, as well as longer follow-up time (1, 7, 12, 14, 15, 22). Additionally, several studies reported that N0 stage was the independent prognostic factor for survival, which, however, has potential bias, given that many patients did not have completely pathological N status (1, 26). Therefore, the N stage was not included in our model.

The major limitation of our study was its retrospective nature. Additionally, there were some potential differences between the two groups, such as the number of pleural nodules and the pathological N stage, leading to the selection bias because there is no consensus on how to choose patients with unexpected PD who can benefit surgical resections. Third, limited resection and no systemic lymphadenectomy could not provide enough information for final pathological staging. Large sample multi-center studies should be conducted in the future to verify the efficacy of surgical procedures in NSCLC patients with PD.

## Conclusion

NSCLC patients with unexpected PD diagnosed intraoperatively potentially benefited from surgical resection of the primary tumor and multidisciplinary therapies. Patients with targeted adjuvant therapy and primary tumor size ≤ 3 cm had a better prognosis. Our data demonstrated that the resection type was not associated with survival differences, which, however, may be influenced by the heterogeneity of the resection group. Further studies on whether the type of surgical resections (sublobar resection vs. lobectomy) affects the survival remain to be determined.

## Data Availability Statement

The raw data supporting the conclusions of this article will be made available by the authors, without undue reservation.

## Ethics Statement

The studies involving human participants were reviewed and approved by the Committee for Ethical Review of Research at Shanghai Chest Hospital and Huadong Hospital. Written informed consent for participation was not required for this study in accordance with the national legislation and the institutional requirements.

## Author Contributions

LF: methodology, software, and writing—original draft. HY: conceptualization and software. KH: data curation. YZ: visualization and investigation. WG: formal analysis. RS: writing—reviewing and editing. FY: validation, writing—reviewing, and editing. HZ: supervision, resources, writing—reviewing, and editing. All authors contributed to the article and approved the submitted version.

## Conflict of Interest

The authors declare that the research was conducted in the absence of any commercial or financial relationships that could be construed as a potential conflict of interest.

## Abbreviations

ALK: anaplastic lymphoma kinase
CI: confidence interval
CT: computed tomography
EGFR: epidermal growth factor receptor
HR: hazard ratio
MPN: malignant pleural nodules
MRI: magnetic resonance imaging
NSCLC: non-small cell lung cancer
OS: median overall survival
PD: pleural dissemination
PET: positron emission tomography
PFS: progression-free survival
PS: performance status
TKI: tyrosine kinase inhibitor
VATS: video-assisted thoracoscopic surgery.
